# Risk Factors for the Completion of the Cold Loop Hysteroscopic Myomectomy in a One-Step Procedure: A Post Hoc Analysis

**DOI:** 10.1155/2018/8429047

**Published:** 2018-05-20

**Authors:** Ivan Mazzon, Alessandro Favilli, Mario Grasso, Stefano Horvath, Vittorio Bini, Gian Carlo Di Renzo, Sandro Gerli

**Affiliations:** ^1^“Arbor Vitae” Endoscopic Centre, Clinica Nuova Villa Claudia, 00191 Rome, Italy; ^2^Department of Obstetrics and Gynecology, University of Perugia, 06156 Perugia, Italy

## Abstract

**Introduction:**

The aim of the study was to analyze which variables influenced the completion of a cold loop hysteroscopic myomectomy in a one-step procedure in a large cohort of patients.

**Materials and Methods:**

A retrospective cohort study of 1434 cold loop resectoscopic myomectomies consecutively performed. The study population was divided into two groups according to the number of procedures needed to accomplish the treatment. Variables influencing the completion of hysteroscopic myomectomy in a one-step procedure were investigated.

**Results:**

A total of 1434 resections were performed and 1690 myomas in total were removed. The procedure was accomplished in a one-step procedure in 1017 patients (83.7%), whereas 198 women (16.3%) needed a multiple-step procedure. The multivariate analysis showed that the size, the number of myomas, and the age of patients were significantly correlated with the risk of a multiple-step procedure. No correlation was revealed with the grading of myomas, parity, and the use of presurgical GnRH-agonist therapy.

**Conclusions:**

In case of multiple fibroids, the intramural development of submucous myomas did not influence the completion of cold loop hysteroscopic myomectomy in a one-step procedure. The size of myomas and the age of patients were significantly correlated with the need to complete the myomectomy in a multiple-step procedure.

## 1. Introduction

Hysteroscopic myomectomy represents the best minimally invasive option for the removal of submucous myomas [1]. Although myomectomy is widely shared and adopted, hysteroscopic treatment of myomas with an intramural extension of 50% or more has always been represented as a challenge for the surgeon, as it influences the possibility of completing the myomectomy in a single-step procedure [1, 2], while increasing the risk for intraoperative complications and repeated surgeries [1, 3, 4]. Moreover, in cases of multiple myomas, the risk is even higher.

As recently reported by Pakrashi, hysteroscopic resection of submucous uterine fibroids should be a simple, well-tolerated, and effective procedure [5]. Several techniques have been reported to date, but the cold loop hysteroscopic myomectomy seems to be the best option, as it allows a safe and complete removal of G1 and G2 myomas in only one-step procedure [1, 6–8]. Different from the classical slicing into the muscular fibers, the cold loop technique allows a safe and complete removal of fibroids characterized by an intramural development, respecting at the same time the surrounding healthy myometrium [1, 6, 8] and the myoma pseudocapsule [9, 10].

The aim of this study was to analyze which patients or myoma characteristics influenced the possibility of completing a cold loop hysteroscopic myomectomy in a single surgical procedure, by assessing a large cohort of patients.

## 2. Materials and Methods

We retrospectively reviewed from our institutional database the series of consecutive patients who underwent cold loop hysteroscopic myomectomy at the “Arbor Vitae” Centre for Endoscopic Gynecology (Clinica Nuova Villa Claudia, Rome, Italy) between January 2003 and December 2010. Institutional Review Board approval was obtained for data collection.

In order to reduce the potential bias due to the retrospective cohort study design, the patients were selected from our institutional database following the same inclusion and exclusion criteria observed in our previous report in which safety and efficacy of the cold loop were investigated [7]. Clinical data only from patients with a histologic confirmation of a myoma were collected. All the patients were studied by ultrasound and outpatient diagnostic hysteroscopy in order to assess the number, the size, and the grading of myomas, the thickness of the free myometrial margin (FMM), and the thickness of the myometrium between the myoma and the perimetrium. A FMM of at least 2 mm was considered adequate for the treatment because, as demonstrated by Casadio et al., it is not a static parameter but grows progressively with each step of the procedure, leading to an increasing margin of safety [11]. Intramural development was catalogued in accordance with the classification of the European Society of Gynaecological Endoscopy: G0: completely endocavitary, pedunculated myoma, with no intramural extension; G1: submucous myoma with less than 50% intramural extension; G2: submucous myoma with more than 50% intramural extension [2].

In case of fibroids greater than 2 cm, three consecutive injections of gonadotropin-releasing hormone (GnRH) agonist (triptorelin 3.75 mg IM) 28 days apart were administered. A new ultrasound scan was carried out after the GnRH-agonist therapy and a 15% reduction in size of submucous myomas was registered.

Before the surgery, informed consent was obtained from all the patients. G1 and G2 fibroids were treated by using the cold loop hysteroscopic myomectomy as previously described [6, 7]. As the cold loop technique was conceived for the treatment of myoma with an intramural component, G0 myomas were enrolled only if present at the same time with G1-G2 fibroids and removed by means of the traditional slicing technique. All the procedures were performed by 4 surgeons with the same experience and skill level, using a 9 mm resectoscope with 0° optical system (HOPKINSII® Karl Storz, Tuttlingen, Germany) and 1.5% glycine for distention of the uterine cavity. Slicing was performed using an electric loop powered by a 100 W monopolar current in pure cutting mode, whereas the enucleation of the intramural component was performed with a nonelectric mechanical loop (Mazzon's cold loops®, Karl Storz, Tuttlingen, Germany). In order to reduce thermal damage of the healthy myometrium as much as possible, coagulation was never used. The continuous and constant irrigation of the distention media was provided by HYDROMAT® (Karl Storz, Tuttlingen, Germany). The intrauterine cavity pressure was set between 90 and 110 mmHg depending on the type of intervention. The liquid balance was monitored by EQUIMAT® (Karl Storz, Tuttlingen, Germany). When the level of absorbed liquid reached 1000 cc or the serum sodium dropped to 125 mEq/l, the myomectomy procedure was interrupted and a second surgery was scheduled. Antibiotic prophylaxis was only administered to patients with specific indications (e.g., cardiac valvulopathies).

Clinical data was collected regarding the characteristics of patients and their obstetric history. Number, size, and grading of myomas as well as the length of surgery (from the introduction of the resectoscope in the uterine cavity until the end of the procedure) were analyzed.

In order to investigate the variables that influenced the need for a multiple-step procedure to completely remove the myomas from the uterine cavity, the study population was divided into two groups: patients who completed the treatment in a single procedure (one-step group, OS) and the patients who accomplished the myomectomy in two or more procedures (multiple-step group, MS). A multivariate analysis was then carried out in order to eliminate confounding factors. In the event of multiple myomas, the grading and size were considered as the mean of the enucleated fibroids.

### 2.1. Statistical Analysis

The Mann–Whitney test was used to compare ordinal and non-normally distributed continuous variables (deviation from Gaussian distribution was checked by using the Shapiro-Wilk test). Categorical data were analyzed by a *χ*2 test with Yate's correction. A multivariate logistic regression model was fit to the prediction of a cold loop hysteroscopic myomectomy in a single-step surgical procedure (coded as yes = 1 and no = 0), incorporating as explanatory variables all the variables that showed a p value ≤ 0.25 in bivariate analysis [12]. The goodness of fit for logistic regression models was checked using the Hosmer and Lemeshow test. Statistical analyses were performed using IBMSPSS ® version 22.0 (IBM Corp., Armonk, NY, USA, 2013). A two-sided p value < 0.05 was considered significant.

## 3. Results

A total of 1215 patients were selected during the study period. There were 1434 resections performed and 1690 myomas in total were removed. The indications for resectoscopic myomectomy were heavy menstrual bleeding in 51.43% and 21.12% of cases in the OS and MS groups, respectively, with infertility (OS 9.3% and MS 3.12%) and intermenstrual spotting (OS 9.82% and MS 0.37%). In 4.84% of cases, the indication was postmenopausal bleeding and in 2.05% of cases there was a thickening of the endometrium, subsequently diagnosed as a myoma.

The cold loop hysteroscopic myomectomy was accomplished in a one-step procedure in 1017 patients (83.7%), whereas 198 women (16.3%) needed a multiple-step procedure. No major complications occurred. Twelve minor intraoperative complications were recorded (0.84%). The characteristics of the patients and myomas are summarized in Table 1.

The mean age was significantly higher in the OS group (p = 0.0001), whereas the number of patients who were given the GnRH agonist was significantly higher in the MS group (p = 0.0001). The size of G1 and G2 myomas was significantly higher in the MS group than in the OS group (0.0001). Multiple fibroids were significantly more numerous in the MS group (p = 0.028). The length of procedures was significantly higher in the MS group than in the OS group (p = 0.0001).

The characteristics of treated myomas in each group are shown in Table 2 according to size and intramural development. The number of removed G1 and G2 myomas greater than 40 mm was significantly higher in the MS group than in the OS group (p = 0.0001). No statistically significant differences were found between the two study groups in the number of G2 and G1 myomas measuring from 20 to 40 mm.

**Figure 1:**
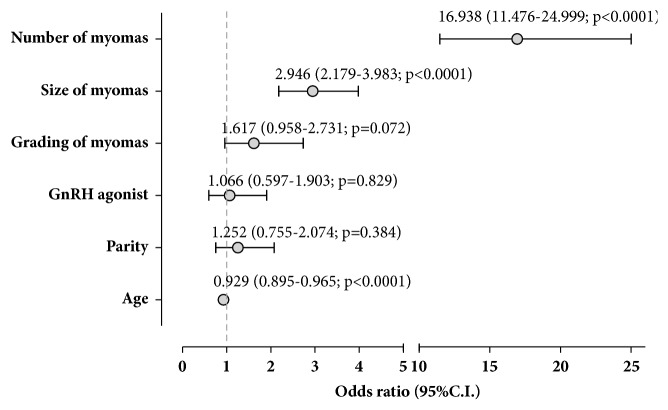
Multiple logistic regression model of variables influencing the multiple-step procedure. In order to scale OR in a more intelligible range, size of myomas is expressed as mean (cm) of all myomas treated in each patient. Grading of myomas is expressed as mean of all myomas treated in each patient. Multivariate logistic regression model was fit to the prediction of a cold loop hysteroscopic myomectomy in a single-step surgical procedure (coded as yes = 1 and no = 0), incorporating as explanatory variables all the variables that showed a p value ≤ 0.25 in bivariate analysis [11]. The goodness of fit for logistic regression models was checked using the Hosmer and Lemeshow test.

Finally, a multivariate analysis was carried out in order to assess which variables significantly influenced the completion of the hysteroscopic myomectomy in one surgical procedure (Figure 1). The size and the number of myomas and the age of patients were significantly correlated with the need to complete the myomectomy in a multiple-step procedure. No correlation was revealed with the grading of myomas, nor with parity or the use of presurgical GnRH-agonist therapy.

## 4. Discussion

The grading of myomas has always been considered a major difficulty in hysteroscopic myomectomy, especially when associated with multiple myomas [3]. In 1993, Wamsteker [2] recommended treating myomas with more than 50% intramural extension only in selected cases, as repeated procedures were usually needed in order to achieve complete resection and resolution of symptoms. Recently, multiple variables and not only the degree of myometrial penetration were considered in the “STEPW classification” proposed by Lasmar et al. [13], to more accurately predict a complete or an incomplete removal of the myoma before treatment. The size of myoma, its topography, the extension of its base, and its penetration into the myometrium all seem to influence the outcome of surgery in a hysteroscopic myomectomy [14]. Nevertheless, we believe that these factors should be correlated with the technique utilized and the number of myomas present in the uterine cavity at the same time.

The matter of treating the intramural extension of myomas in order to resolve the related clinical symptoms has been stressed over the last three decades and with this purpose several techniques have been described [1]. Some have been conceived as a “two-step procedure” [15, 16], but they are burdened with a double anesthetic and surgical risk for the patients; other techniques were instead conceived as a “one-step procedure”, with the aim of accomplishing the myomectomy in only one surgical session [17–21]. In the latter case, the skill level of the surgeon and the characteristics of the myoma may determine the success of the hysteroscopic myomectomy. But when we deal with cold loop hysteroscopic myomectomy, which is a highly recognized procedure useful to remove single or multiple myomas respecting the pseudocapsule [22–25], we may consider that myoma progressively moves during surgery from the inner myometrium to the endocavitary region.

The cold loop technique allows treating the intramural component of G1 and G2 myomas by the blunt dissection of the fibroconnectival bridges which anchor the myoma to the pseudocapsule [22, 26]. In this way, the intramural portion of the myoma slides in the uterine cavity becoming an intracavitary lesion, easy to treat by classical slicing. Therefore, the intramural component of myoma loses its importance and the most difficult phase of the procedure is dependent on the size rather than on the myoma grading.

Although a correct comparison is difficult and risks are being somewhat arbitrary, our results seem to be in agreement with the available literature [1–3, 14, 27–29] as well as with the results previously reported by Leone using the cold loop technique [30]. Indeed, in our series 1017 patients (83.7%) accomplished the treatment in a one-step procedure. Therefore, differently from the intramural slicing, which inevitably damages the surrounding healthy myometrium because of the fibers cutting and the thermal damage, the cold loop myomectomy enables an effective treatment of G1 and G2 myomas, with a low rate of intraoperative complications and virtually eliminating uterine perforation with thermal loops [6, 7].

In a previous study, we evaluated the chance of completing the treatment in a single step with the cold loop hysteroscopic myomectomy and, in order to avoid confounding factors, we selected a cohort of patients with only a single myoma. The results showed that the size, the grading of myomas, and the age of patients were the independent variables that influenced the completion of procedures in only one surgical step [8]. Different from the aforementioned investigation, in the present study, we selected all the patients from our institutional database, including those treated with multiple myomas and the grading of myomas seems to lose its important role. Indeed, the logistic regression analysis showed a minor importance of the intramural development and a significant role of number and size of myomas and the age of patients (P = 0.0001). We speculate that these contradictory results may be justified by the inclusion of patients with multiple myomas. The analysis of surgical procedures in two different populations of patients cannot be considered in the same way. In the first study [8], we provided the information to the surgeon that, if a single myoma has to be removed with this technique, a special care should be given to the size and the grading of the fibroid. This study, on the contrary, provides different recommendations in case of patients with multiple myomas. As predictable, the surgeon should be aware that size and number are related to a multiple-step procedure, but not the intramural component of fibroids.

Concerning the myomas size, it should be underlined that, with increasing diameter, the volume of myoma increases much faster (to the third power). As reported by Emanuel, this is of great influence on the ultimate surgery time that is necessary for the complete removal of myoma by hysteroscopic techniques [31]. In our series, the G2 myomas in MS group were double in volume with respect to the G2 in OS group. Indeed, a multivariate analysis demonstrated that the risk of a multiple-step procedure increased by about 3 times for each centimeter of myoma (O.R. 2.946; p < 0.0001).

In our experience, the age of patients was inversely correlated with a multiple-step procedure. We speculate that a possible explanation for this phenomenon is myometrial dysfunction, which is detectable in women over the age of 40 [32–34]. Indeed, both blood supply and the absorption of distention media are decreased because of reduced uterine vascularization, which is characteristic of women approaching menopause.

The GnRHa administration before surgery was not correlated with multiple-step procedure. We speculate that this issue could be a bias due to the retrospective analysis of data. Indeed, the lack of estrogens induced by GnRHa administration, as clearly demonstrated by De Falco et al., affects both the fibroid and the uterus compacting the tissue including the pseudocapsule, increasing the difficulty of the myoma dissection from the surrounding myometrium [35]. Recently, we published a RCT with the aim of evaluating the intraoperative effects of GnRHa pretreatment in patients undergoing cold loop hysteroscopic myomectomy. The results showed that the preoperative GnRHa administration did not facilitate the completion of cold loop hysteroscopic myomectomy in a single surgical procedure in G2 myomas and was correlated with a longer duration of the surgery. No significant benefits were found for G0 and G1 myomas [36].

The four expert surgeons, with a high experience in cold loop myomectomy, could represent the main limitation of this retrospective study. Indeed, the cold loop technique requires an adequate surgical experience [1], yet we believe that, as already demonstrated [6, 7, 30, 37], with an acceptable learning curve it is possible to safely carry out a satisfying procedure with excellent outcomes.

## 5. Conclusions

In conclusion, we can affirm that, in case of multiple G1 and G2 myomas, the intramural development did not influence the completion of the cold loop hysteroscopic myomectomy in a single procedure. Nevertheless, the size of myomas and the age of patients were significantly correlated with the need to complete the myomectomy in a multiple-step procedure. In the presence of these risk factors, the option of a second-step procedure should be taken into consideration by the surgeon.

The low rate of complications, the number of treatments accomplished in a single procedure, even in case of multiple myomas, and the fact that myomectomies were performed by 4 different surgeons point out that cold loop technique is not only safe and effective, but also repeatable.

## Figures and Tables

**Table 1:** Characteristics of patients.

	One-step group (OS)	Multiple-step group (MS)	Total	p
Patients	1017 (83.7)	198 (16.3)	1215	-
Procedures	1017 (70.92)	417 (29.08)	1434	-

Age (years)^*∗*^	43.13 ± 8.24	40.42 ± 6.08	42.69 ± 7.99	0.0001
Nulliparous	640 (62.9)	135 (68.2)	775	0.216
Pluriparous	377 (37.1)	63 (31.8)	440	
Previous cesarean section	159 (15.6)	26 (13.1)	185 (15.2)	0.415
GnRH agonist	445 (43.8)	134 (67.7)	579 (47.6)	0.0001
Length of procedures (minutes)^*∗*^	14.16 ± 9.24	19.08 ± 14.16	16 ± 11.05	0.0001

G0	34 (2.89)	13 (2.54)	47 (2.78)	0.75
G1	753 (63.92)	285 (55.66)	1038 (61.42)	0.0016
G2	391 (33.19)	214 (41.8)	605 (35.8)	0.0008

G0 size (mm)^*∗*^	18.03 ± 7.82	18.31 ± 8.45	18.11 ± 7.9	0.865
G1 size (mm)^*∗*^	20.94 ± 8.79	24.12 ± 11.21	21.81 ± 9.62	0.0001
G2 size (mm)^*∗*^	22.49 ± 9.11	28.6 ± 10.5	24.66 ± 10.05	0.0001

Single myoma procedures	901(88.6)	351 (84.2)	1252 (87.3)	0.028
Multiple myomas procedures	116 (11.4)	66 (15.8)	182 (12.7)	
Total myomas removed	1178 (69.7)	512 (30.3)	1690	0.023

Data as reported as n (%) p = *χ*2 test with Yate's correction

*∗* mean ± SD p = Mann–Whitney test.

**Table 2:** Number of myomas treated according to the size (mm).

	G0			G1			G2		
One-step procedures	>40	40-20	<20	>40	40-20	<20	>40	40-20	<20
	0 (0)	18 (52.9)	16 (47.1)	12 (1.6)	471 (62.5)	270 (35.9)	13 (3.3)	260 (66.5)	118 (30.2)

Multiple-step procedures	>40	40-20	<20	>40	40-20	<20	>40	40-20	<20
	0 (0)	7 (53.8)	6 (46.2)	20 (7)	172 (60.4)	93 (32.6)	30 (14)	149 (69.6)	35 (16.4)

p	0	0.955	0.955	0.0001	0.562	0.368	0.0001	0.486	0.0003

Data are reported as n (%)

p = *χ*2 test with Yate's correction.
